# Impact of Cefazolin Shortage on Clinical Outcomes of Adult Patients with Bacteremia Caused by Methicillin-Susceptible *Staphylococcus aureus* in a Tertiary Care University Hospital

**DOI:** 10.3390/antibiotics10101247

**Published:** 2021-10-14

**Authors:** Atsushi Uda, Kenichiro Onuma, Katsumi Shigemura, Koichi Kitagawa, Yonmin Yan, Kayo Osawa, Ikuko Yano, Takayuki Miyara

**Affiliations:** 1 Department of Infection Control and Prevention, Kobe University Hospital, Kobe 650-0017, Japan; onumak@med.kobe-u.ac.jp (K.O.); katsumi@med.kobe-u.ac.jp (K.S.); miyarat@med.kobe-u.ac.jp (T.M.); 2 Department of Pharmacy, Kobe University Hospital, Kobe 650-0017, Japan; iyano@med.kobe-u.ac.jp; 3 Department of Clinical Laboratory, Kobe University Hospital, Kobe 650-0017, Japan; 4 Division of Infectious Diseases, Department of Public Health, Kobe University Graduate School of Health Sciences, Kobe 654-0142, Japan; ko1.kitgwa@gmail.com; 5 Division of Urology, Kobe University Graduate School of Medicine, Kobe 650-0017, Japan; yym1112@gmail.com; 6 Division of Advanced Medical Science, Kobe University Graduate School of Science, Technology and Innovation, Kobe 657-8501, Japan; 7 Department of Medical Technology, Kobe Tokiwa University, Kobe 653-0838, Japan; k-ohsawa@kobe-tokiwa.ac.jp

**Keywords:** antimicrobial shortage, cefazolin, bacteremia, methicillin-susceptible *Staphylococcus aureus*

## Abstract

Cefazolin is an essential antibiotic used for treating bacteremia; in particular, it is recommended as a first-line agent for infections caused by methicillin-susceptible *Staphylococcus* *aureus* (MSSA). In March 2019, problems with a major antibiotic supplier caused a critical shortage of cefazolin in Japan; however, the impact of the cefazolin shortage on clinical outcomes remains unknown. This study aimed to evaluate the effect of the cefazolin shortage in patients with MSSA bacteremia. Data from 75 patients were compared between the pre-shortage (March 2018–January 2019, n = 39) and post-shortage (March 2019–January 2020, n = 36) periods. There were no significant differences in the demographic characteristics between the two groups, and the cefazolin shortage did not worsen clinical outcomes such as adverse drug reactions, treatment failure, and 30-day mortality. In the post-shortage group, ampicillin/sulbactam and benzylpenicillin were more frequently administered as alternative antibiotics for empirical and definitive therapy (10% vs. 31%, *p* = 0.042; 0% vs. 19%, *p* = 0.004, respectively). Multivariate analysis revealed that the broad-spectrum antibiotics for definitive therapy, such as antipseudomonal penicillin, were associated with treatment failure in patients with MSSA bacteremia (OR = 17, *p* = 0.003). Hence, narrow-spectrum antibiotics should be prescribed for MSSA bacteremia as alternatives during a cefazolin shortage.

## 1. Introduction

Methicillin-susceptible *Staphylococcus aureus* (MSSA) is a common cause of hospital-acquired infections and is associated with poor clinical outcomes [1]. According to clinical guidelines, anti-staphylococcal penicillins (such as nafcillin, oxacillin, cloxacillin, and flucloxacillin) and the cephalosporin cefazolin should be used for treating MSSA [2,3,4]. Anti-staphylococcal penicillins are unavailable locally in Japan. Therefore, cefazolin, which is a narrow-spectrum first-generation cephalosporin, is used as the first choice for treating MSSA bacteremia [5]. Cefazolin is associated with better outcomes for MSSA infections than vancomycin, which is effective against most Gram-positive bacteria including methicillin-resistant *S. aureus* (MRSA) [6,7].

Critical antimicrobial shortages pose detrimental healthcare risks worldwide [8]. In March 2019, Japan experienced a cefazolin shortage due to a supplier problem [9,10]. The cefazolin shortage affected infectious disease treatment in many hospitals because cefazolin is one of the most administered parenteral agents in Japan [11]. The Ministry of Health, Labour and Welfare of Japan issued a notice regarding the use of alternative antibiotics for treating specific infectious diseases [12]. Blood is a sterile specimen; if bacteria are detected in blood culture, it is highly possible that the isolated bacteria are the causative microorganism of the infection. This list includes bacteremia with MSSA listed as the only causative pathogen [12]. Therefore, we focused on bacteremia caused by MSSA in this study. Alternative antibiotics listed for treating MSSA bacteremia are ampicillin/sulbactam, third-generation cephalosporins (cefotaxime and ceftriaxone), and anti-MRSA agents (vancomycin and daptomycin) [12]. A previous study reported that the antimicrobial shortage was associated with an increased prescription of broad-spectrum antibiotics as alternative antibiotics [13]. Although alternative antibiotic use may have a negative effect [14], the effect of a cefazolin shortage on the treatment of patients with bacteremia remains unclear. Thus, this study aimed to evaluate the effect of a cefazolin shortage on the clinical outcomes of patients with bacteremia caused by MSSA at a university hospital.

## 2. Results

During the study period, we extracted data on all patients with bacteremia caused by pathogens including MSSA. After excluding four patients aged <18 years and 16 patients infected with polymicrobial strains, 75 patients were included in this study (pre-shortage group, n = 39; post-shortage group, n = 36). The prescribed antibiotics in this study were as follows: penicillins except for antipseudomonal agents—benzylpenicillin, ampicillin, and ampicillin/sulbactam; antipseudomonal penicillin—piperacillin/tazobactam; first-generation cephalosporin—cefazolin; second-generation cephalosporin—cefotiam; carbapenem—meropenem; and anti-MRSA agents—vancomycin, daptomycin, and linezolid.

The demographic characteristics of each group are summarized in Table 1. No significant differences were observed between the two groups with respect to sex, age, hospitalization ward, hospital stay before bacteremia onset, previous immunosuppression, recent surgery, hemodialysis, invasive device use, vasopressor use, quick sequential organ failure assessment (qSOFA) score ≥2, or altered mental status. The most common primary source of infection was a catheter-related bloodstream infection (n = 22, 29%), followed by a skin and soft tissue infection (n = 13, 17%), bone infection (n = 8, 11%), infectious endocarditis (n = 6, 8.0%), respiratory tract infection (n = 4, 5.3%), and febrile neutropenia (n = 3, 4.0%). The strains that were susceptible to ampicillin were detected in 44% (17/39) and 56% (20/36) of patients in the pre- and post-shortage groups, respectively. None of the strains were resistant to all other antibiotics such as ampicillin/clavulanate, cefazolin, cefotiam, and vancomycin. In empirical therapy, the prescriptions of penicillins except for the antipseudomonal agents ampicillin/sulbactam, were more common in the post-shortage period than in the pre-shortage period (31% vs. 10%, *p* = 0.042). Cefazolin had a higher prescription rate in the pre-shortage group than in the post-shortage group (31% vs. 8%, *p* = 0.021).

Table 2 shows the clinical outcomes of patients with MSSA bacteremia. No significant differences were observed between the pre- and post-shortage groups with respect to the time to fever resolution, time to white blood cell count normalization, time to negative blood culture results, rates of persistent bacteremia, alternative antibiotic therapy for presumed treatment failure, time to initial antibiotic therapy, total duration of antibiotic therapy, hospital length after the onset of bacteremia until discharge, readmission within 90 days after discharge, treatment failure, daily antimicrobial cost, or adverse drug reactions (*Clostridioides difficile* infection, diarrhea, and skin rash). In definitive therapy, benzylpenicillin was more frequently prescribed in the post-shortage group than in the pre-shortage group (19% vs. 0%, *p* = 0.004). The percentage of patients receiving cefazolin for definitive therapy was significantly lower in the post-shortage group than in the pre-shortage group (53% vs. 82%, *p* = 0.014).

In this study cohort, the treatment failure rate was 24% (18/75). Table 3 shows the risk factors associated with treatment failure in patients with MSSA bacteremia. Vasopressor use altered mental status, and piperacillin/tazobactam prescriptions for definitive therapy were associated more frequently with treatment failure. These variables were included in the multivariate logistic regression analysis. Piperacillin/tazobactam for definitive therapy (OR = 17, *p* = 0.003) and altered mental status (OR = 12.7, *p* = 0.004) were identified as independent risk factors for treatment failure.

**Figure 1:**
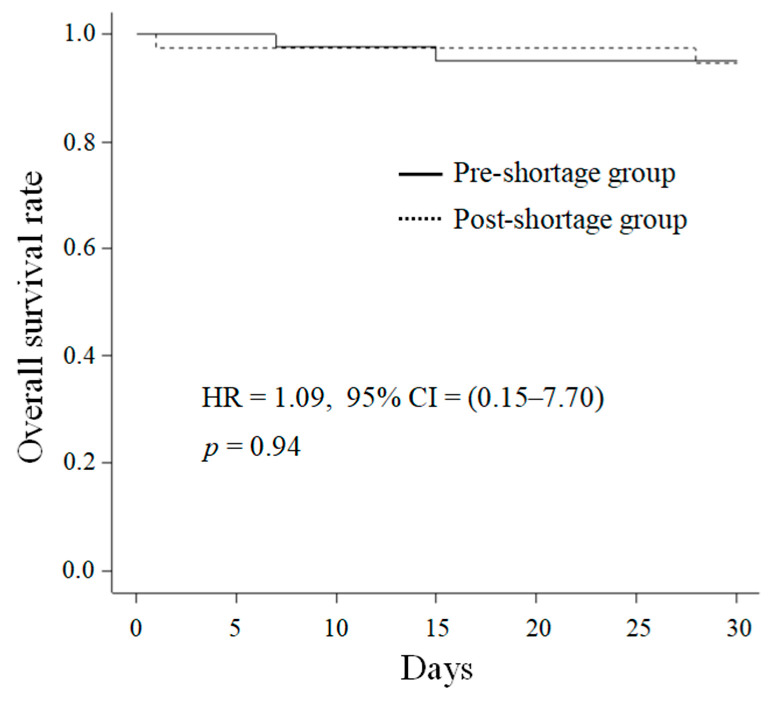
Kaplan–Meier survival curves of pre- and post-shortage groups.

Figure 1 shows the Kaplan–Meier curve of the survival rates for both the pre- and post-shortage groups. Two patients in each group died within 30 days after the onset of bacteremia. The 30-day mortality rates were similar between the two groups (5.1% vs. 5.6%, *p* = 1.0).

## 3. Discussion

We investigated the effect of a cefazolin shortage on the treatment of patients with MSSA bacteremia. In our hospital, while the use of cefazolin decreased, the use of penicillins except for antipseudomonal agents, increased during the cefazolin shortage period. Furthermore, no significant differences were observed between the pre- and post-shortage groups with respect to clinical outcomes. We identified that the use of an antipseudomonal agent, piperacillin/tazobactam, as definitive therapy and altered mental status at the onset of bacteremia were independent risk factors for treatment failure for MSSA bacteremia. The cefazolin shortage was associated with an increased use of penicillins except for antipseudomonal agents, as alternative agents for treating MSSA bacteremia with no deterioration in clinical outcomes.

Cefazolin acts against both Gram-positive and Gram-negative bacteria and is recommended as an ACCESS group antibiotic on the World Health Organization (WHO) Model List of Essential Medicines [15]. Cefazolin, which is superior to vancomycin for treating MSSA bacteremia [6,7], is used as the first-choice agent to treat MSSA because anti-staphylococcal penicillins (nafcillin, oxacillin, cloxacillin, and flucloxacillin) are not approved and are, therefore, unavailable for clinical use in Japan. In March 2019, Nichi-Iko Pharmaceutical Co., Ltd., a major supplier in Japan, announced that the cefazolin supply was suspended because of manufacturing problems [9]. In response to the shortage of cefazolin, the Japanese Ministry of Health, Labour and Welfare listed broad-spectrum antibiotics as an alternative therapy for infectious diseases caused by MSSA [12]. However, in this study, these listed antibiotics were not primarily used as an alternative for the definitive treatment of MSSA bacteremia.

An antimicrobial shortage may pose a serious threat to patient outcomes. A previous study reported that an antimicrobial shortage was associated with an increase in cost and a decrease in the susceptibility of Gram-negative bacteria [14]. Broad-spectrum antibiotics are associated with an increased risk of acquiring and developing resistant bacteria [16]; using narrow-spectrum antibiotics is cost-effective and should be encouraged via antimicrobial stewardship programs [17]. Although first-generation cephalosporins have coverage against most Gram-positive cocci, alternative broad-spectrum agents such as second- and third-generation cephalosporins have weaker effects on Gram-positive cocci than first-generation cephalosporins. A recent retrospective study reported that alternative antimicrobial prophylaxis antibiotics, which comprised broad-spectrum antibiotics, were associated with an increased risk of surgical site infection in spine surgery during the cefazolin shortage in Japan [18]. Furthermore, the piperacillin/tazobactam shortage in the USA increased meropenem prescriptions despite interventions via antimicrobial stewardship programs [13,19]. The cefepime shortage also led to increased consumption of piperacillin/tazobactam and meropenem, as well as higher overall costs and decreases in susceptibility to *Pseudomonas aeruginosa* [14]. These studies show that antimicrobial shortages can lead to the increased use of alternative broad-spectrum antimicrobials and worse clinical outcomes. However, no inferior changes were observed in the antimicrobial therapy and patient outcomes in the current study.

In this study, to restrict cefazolin use, cefazolin was prescribed specifically to patients that were vulnerable to severe illness or those with bacteremia caused by penicillin-resistant *S. aureus*. Previous cohort studies reported that penicillins could be used as alternative agents for the definitive treatment of penicillin-susceptible bacteremia in patients with stable conditions [20,21]. We found that penicillins were mainly prescribed to patients with bacteremia due to penicillin-susceptible *S. aureus*, leading to a significant increase in penicillin use. We also observed a significant increase in the empirical use of ampicillin/sulbactam during the shortage period. Although 50.7% of MSSA strains were resistant to ampicillin, all of the strains were susceptible to ampicillin/sulbactam. When suspecting infections with pathogens including MSSA, ampicillin/sulbactam, rather than benzylpenicillin or ampicillin, was chosen as the empirical antimicrobial agent. However, a recent retrospective cohort study reported that benzylpenicillin had potential benefits for treating bloodstream infections that were caused by penicillin-susceptible *S. aureus* compared with flucloxacillin treatment [21]. Consistent with this study, benzylpenicillin was more frequently prescribed according to microbiological test results. 

Our findings also showed no adverse clinical outcomes after the cefazolin shortage. Although the incidence of *C. difficile* infection (CDI) increased during the piperacillin/tazobactam shortage [19,22], we found no significant difference in the incidence of CDI and diarrhea between the two groups. Our findings may be clinically plausible because penicillins have been reported to be associated with lower CDI episodes than cephalosporins [23], and penicillins were more frequently prescribed during the cefazolin shortage. Meanwhile, penicillins are associated with a higher incidence rate of antimicrobial allergies than cephalosporins [24]. Approximately 8% of patients have a history of penicillin allergies, whereas cephalosporin allergies have been reported to be as low as 1% [25]. Although the use of penicillins increased for treating MSSA bacteremia, persistent skin rashes were observed in one patient in each group. Among four patients who died within 30 days of bacteremia onset, one died owing to malignancy and one died owing to pneumonia in each group, indicating no association between death and antimicrobial use.

We identified that piperacillin/tazobactam for definitive therapy was associated with treatment failure for MSSA bacteremia. According to a retrospective cohort study, when comparing piperacillin/tazobactam with cefazolin for treating bacteremia due to MSSA, the mortality rate was higher in the piperacillin/tazobactam group [26]. These findings suggest that it may not be effective in the treatment of MSSA bacteremia. Furthermore, our results showed that an altered mental status was a risk factor associated with treatment failure. A previous study reported that the risk factors for mortality owing to *S. aureus* were Charlson’s weighted index of comorbidity score > 5, previous hospitalization, and altered mental status [27]. An altered mental status is common in patients with sepsis and is associated with higher mortality [28]. Our findings are consistent with those of previous studies [27,28].

Our study had some limitations. This was a single-center retrospective study with a small number of patients. Our enrolled patients only had MSSA bacteremia. To determine the effect of a cefazolin shortage on antimicrobial prescription and patient outcomes in an institute, we must broadly investigate the characteristics of patients with other bacteremia treated with cefazolin as the first choice.

## 4. Methods

### 4.1. Setting and Patients

At Kobe University Hospital, the prescription of cefazolin was restricted from March 2019 to January 2020 because a major antibiotic supplier caused a national shortage of cefazolin in Japan. This observational study was retrospectively conducted between the pre-shortage (March 2018–January 2019) and post-shortage (March 2019–January 2020) periods. Details of antimicrobial use and patient data were obtained from electronic medical records. Patients with bacteremia were characterized based on the identification of at least one MSSA in blood culture, and patients who received intravenous antibiotics were included in this study. We excluded patients aged <18 years or those with a polymicrobial blood culture.

### 4.2. Definition

We classified penicillins available in our hospital into the penicillins except for antipseudomonal agents (benzylpenicillin, ampicillin, and ampicillin/sulbactam) and antipseudomonal penicillins (piperacillin and piperacillin/tazobactam). The hospitalization ward, hemodialysis, invasive device use, vasopressor use, qSOFA score, and altered mental status were evaluated for bacteremia onset. Data on previous immunosuppression and recent surgery 30 days before bacteremia onset were collected. The antimicrobial susceptibility testing of *S. aureus* was performed using the MicroScan WalkAway 96 Plus (Beckman Coulter, Inc., Brea, CA, USA). We defined empirical antibiotic therapy as antibiotics prescribed before revealing the susceptibility data, approximately 3 days after blood culture collection. Definitive therapy was defined as antibiotic therapy prescribed according to the microbiological data of *S. aureus* detected from the blood culture. The time to fever resolution was calculated as the number of days from bacteremia onset until the blood temperature dropped below 37.5 °C. The time to white blood cell normalization was defined as the number of days from bacteremia onset until the white blood cell count dropped below 8600/μL. The time to detect negative blood culture was defined as the number of days after the onset of bacteremia in which a negative culture was detected. Persistent bacteremia was defined as positive blood cultures for MSSA that persisted for ≥7 days. Alternative antibiotic therapy for presumed treatment failure was defined as a change in antibiotics owing to treatment failure. The time to initial antibiotic therapy was defined as the number of days from the onset of bacteremia to the start of the initial antibiotic therapy. The total duration of antibiotic use was defined as the number of days of antibiotic prescription. The hospital length after the onset of bacteremia was defined as the number of days from hospitalization until discharge. Readmission within 90 days after discharge was defined as readmission to the hospital for any cause. Treatment failure was defined as escalation to broad-spectrum antibiotics owing to deteriorating condition, no defervescence within 14 days after bacteremia onset, or death within 30 days after bacteremia onset. We calculated the cost of antimicrobial agents per day during antibiotic therapy for MSSA bacteremia by multiplying the drug price per dose by the total number of doses administered and dividing the product by the total number of days of antibiotic therapy. All costs are expressed in U.S. dollars (USD; exchange rate, USD1 = 110.0 yen as of 1 September 2021). Adverse drug reactions were observed within 30 days of bacteremia onset. To isolate *C. difficile*, fecal suspensions treated with 99% ethanol were cultured on cycloserine-cefoxitin mannitol agar (CCMA Media EX) (Nissui Pharmaceutical, Co., Ltd., Tokyo, Japan) and incubated anaerobically for 48 h at 37 °C. The toxigenicity of isolates was determined using immunoassay test C. DIFF QUIK CHEK COMPLETE (Abbott Japan LLC, Tokyo, Japan). 

### 4.3. Statistical Analysis

Continuous and categorical variables were expressed as medians with interquartile ranges (IQRs) and frequency counts with percentages, respectively. Non-parametric variables were analyzed using the Mann–Whitney U-test. Analyses of categorical variables were conducted using the chi-square test or Fisher’s exact test. Independent predictors of treatment failure were identified using multivariate logistic regression analyses. We identified the potential variables for the multivariate logistic regression analysis with a *p*-value < 0.05 according to a univariate analysis. The 30-day mortality was analyzed using Kaplan–Meier analysis, and HR and 95% CI were estimated using multivariate Cox proportional hazard regression models. Statistical significance was set at *p* values < 0.05. All parameters were analyzed using EZR (Saitama Medical Center, Jichi Medical University, Saitama, Japan).

## 5. Conclusions

An antimicrobial shortage affected the trends of antimicrobial prescriptions for treating MSSA bacteremia. Due to the cefazolin shortage, the use of penicillins except for antipseudomonal agents, increased as an alternative agent for treating MSSA bacteremia, with no changes in clinical outcomes including adverse drug reactions, treatment failure, and 30-day mortality. We also identified the antipseudomonal penicillin piperacillin/tazobactam for definitive therapy and altered mental status as independent risk factors for treatment failure due to MSSA bacteremia. These findings demonstrate that the prescription of narrow-spectrum antibiotics should be given as an alternative in the face of a shortage of cefazolin.

## Figures and Tables

**Table 1:** Comparison of demographic characteristics of patients with MSSA bacteremia.

	Pre-Shortage Group (n = 39)	Post-Shortage Group (n = 36)	*p*
Male sex, n (%)	27 (69)	23 (64)	0.81
Age, median years (IQR)	69 (56–80)	69 (54–75)	0.54
Hospitalization ward at the onset of bacteremia, n (%)			
Medical ward	22 (56)	13 (36)	0.13
Surgical ward	14 (36)	15 (42)	0.78
Intensive care unit	3 (8)	8 (22)	0.11
Hospital stay before the onset of bacteremia, median days (IQR)	3 (0–20)	3 (0–21)	0.63
Immunosuppression, n (%)			
Immunosuppressive treatment	1 (3)	4 (11)	0.19
Corticosteroid treatment	11 (28)	7 (19)	0.54
Chemotherapy	7 (18)	2 (6)	0.16
Recent surgery, n (%)	4 (10)	4 (11)	1
Hemodialysis, n (%)	3 (8)	5 (14)	0.47
Invasive devices, n (%)			
Central venous catheter	8 (21)	13 (36)	0.21
Urinary tract infections	6 (15)	6 (17)	1
Mechanical ventilation	1 (3)	1 (3)	1
Vasopressor use, n (%)	7 (18)	6 (17)	1
qSOFA score ≥2, n (%)	12 (31)	14 (39)	0.62
Altered mental status, n (%)	4 (10)	7 (19)	0.34
Source of bacteremia, n (%)			
Catheter-related bloodstream infection	10 (26)	12 (33)	0.63
Skin and soft tissue infection	7 (18)	6 (17)	1
Bone infection	3 (8)	5 (14)	0.47
Infectious endocarditis	4 (10)	2 (6)	0.68
Respiratory tract infection	2 (5)	2 (6)	1
Febrile neutropenia	3 (8)	0 (0)	0.24
Unknown	4 (10)	6 (17)	0.51
Others	5 (13)	2 (6)	0.43
Susceptibility to ampicillin, n (%)	17 (44)	20 (56)	0.42
Empirical antibiotic therapy, n (%)			
Ampicillin/sulbactam	4 (10)	11 (31)	0.042
Piperacillin/tazobactam	8 (21)	6 (17)	0.9
Cefazolin	12 (31)	3 (8)	0.021
Cefotiam	0 (0)	3 (8)	0.11
Meropenem	1 (3)	2 (6)	0.61
Vancomycin	19 (49)	13 (36)	0.071
Daptomycin	3 (8)	1 (3)	0.62
Linezolid	0 (0)	1 (3)	0.48

MSSA: methicillin-susceptible *Staphylococcus aureus*; IQR: interquartile range; qSOFA: quick sequential organ failure assessment.

**Table 2:** Clinical outcomes of patients with MSSA bacteremia.

	Pre-Shortage Group (n = 39)	Post-Shortage Group (n = 36)	*p*
Time to fever resolution, median days (IQR)	2 (1–4)	3 (1–4)	0.98
Time to WBC count normalization, median days (IQR)	4 (0–13)	2 (0–7)	0.28
Time to detect negative blood culture results, median days (IQR)	5 (3–7)	4 (3–5)	0.07
Persistent bacteremia (≥7 days), n (%)	3 (8)	0 (0)	0.24
Alternative antibiotic therapy for presumed treatment failure, n (%)	6 (15)	10 (28)	0.31
Time to initial antibiotic therapy, median days (IQR)	0 (0–1)	0 (0–0)	0.95
Total duration of antibiotic therapy, median days (IQR)	22 (16–35)	17 (15–33)	0.46
Hospital length after the onset of bacteremia until discharge, median days (IQR)	35 (22–59)	32 (19–46)	0.68
Readmission within 90 days after discharge, n (%)	7 (18)	7 (19)	1
Treatment failure, n (%)	7 (18)	11 (31)	0.31
Daily antimicrobial cost, median USD (IQR)	8 (5–17)	11 (4–20)	0.46
Adverse drug reactions, n (%)			
*Clostridioides difficile* infection	1 (3)	1 (3)	1
Diarrhea	6 (15)	5 (14)	1
Skin rash	1 (3)	1 (3)	1
Definitive antibiotic therapy, n (%)			
Benzylpenicillin	0 (0)	7 (19)	0.004
Ampicillin	2 (5)	6 (17)	0.14
Ampicillin/sulbactam	0 (0)	2 (6)	0.23
Piperacillin/tazobactam	2 (5)	7 (19)	0.08
Cefazolin	32 (82)	19 (53)	0.014
Cefotiam	0 (0)	1 (3)	0.48
Vancomycin	5 (13)	3 (8)	0.71
Linezolid	0 (0)	1 (3)	0.48

MSSA: methicillin-susceptible *Staphylococcus aureus*; IQR: interquartile range; WBC: white blood cell.

**Table 3:** Factors associated with treatment failure for MSSA bacteremia.

	Treatment Success (n = 57)	Treatment Failure (n = 18)	*p*	Adjusted OR (95% CI)	*p*
Male sex, n (%)	36 (63)	14 (78)	0.39		
Age, median years (IQR)	69 (56–76)	69 (56–80)	0.44		
Admission to intensive care unit, n (%)	7 (12)	4 (22)	0.51		
Vasopressor use, n (%)	6 (11)	7 (39)	0.016	1.67 (0.30–9.28)	0.56
Altered mental status, n (%)	3 (5)	8 (44)	<0.001	12.7 (2.24–71.9)	0.004
qSOFA score ≥2, n (%)	17 (30)	9 (50)	0.19		
Ampicillin-resistant MSSA, n (%)	30 (53)	8 (44)	0.74		
Unknown source of bacteremia, n (%)	8 (14)	2 (11)	1		
Shortage of cefazolin, n (%)	32 (56)	11 (61)	0.31		
Empirical antibiotic therapy, n (%)					
Ampicillin/sulbactam	11 (9)	4 (22)	0.75		
Piperacillin/tazobactam	11 (19)	3 (17)	1		
Cefazolin	12 (21)	3 (17)	0.25		
Vancomycin	26 (46)	6 (33)	0.23		
Definitive antibiotic therapy, n (%)					
Piperacillin/tazobactam	2 (4)	7 (39)	<0.001	17 (2.61–111)	0.003
Cefazolin	41 (72)	10 (56)	0.31		
Vancomycin	5 (9)	3 (17)	0.39		

MSSA: methicillin-susceptible *Staphylococcus aureus*; IQR: interquartile range; qSOFA: quick sequential organ failure assessment.

## Data Availability

Data sharing is not applicable to this article.
